# Do gender differences in audio-visual benefit and visual influence in audio-visual speech perception emerge with age?

**DOI:** 10.3389/fpsyg.2015.01014

**Published:** 2015-07-16

**Authors:** Magnus Alm, Dawn Behne

**Affiliations:** Department of Psychology, Norwegian University of Science and TechnologyTrondheim, Norway

**Keywords:** audio-visual speech, age related audio-visual experience, gender differences, visual influence, AV benefit

## Abstract

Gender and age have been found to affect adults’ audio-visual (AV) speech perception. However, research on adult aging focuses on adults over 60 years, who have an increasing likelihood for cognitive and sensory decline, which may confound positive effects of age-related AV-experience and its interaction with gender. Observed age and gender differences in AV speech perception may also depend on measurement sensitivity and AV task difficulty. Consequently both AV benefit and visual influence were used to measure visual contribution for gender-balanced groups of young (20–30 years) and middle-aged adults (50–60 years) with task difficulty varied using AV syllables from different talkers in alternative auditory backgrounds. Females had better speech-reading performance than males. Whereas no gender differences in AV benefit or visual influence were observed for young adults, visually influenced responses were significantly greater for middle-aged females than middle-aged males. That speech-reading performance did not influence AV benefit may be explained by visual speech extraction and AV integration constituting independent abilities. Contrastingly, the gender difference in visually influenced responses in middle adulthood may reflect an experience-related shift in females’ general AV perceptual strategy. Although young females’ speech-reading proficiency may not readily contribute to greater visual influence, between young and middle-adulthood recurrent confirmation of the contribution of visual cues induced by speech-reading proficiency may gradually shift females AV perceptual strategy toward more visually dominated responses.

## Introduction

Behavioral research has reported gender (e.g., Dancer et al., 1994; Öhrström and Traunmüller, 2004; Irwin et al., 2006; Strelnikov et al., 2009) and age (e.g., Sommers et al., 2005; Winneke and Phillips, 2011) differences in the utilization of visual speech. Females have been shown to be better speech-readers than males (e.g., Johnson et al., 1988; Dancer et al., 1994; Watson et al., 1996; Strelnikov et al., 2009) as well as being more influenced by the visual signal in audio-visual (AV) speech perception (Aloufy et al., 1996; Öhrström and Traunmüller, 2004; Irwin et al., 2006). In addition, neuroanatomical studies have indicated that when presented with visual speech, females have a stronger activation in brain areas associated with speech perception than males (Ruytjens et al., 2006, 2007). Neuroanatomical studies have also suggested gender differences in lateralization of speech processing (e.g., Shaywitz et al., 1995; Jaeger et al., 1998), where females have a more bilateral processing for word recognition (e.g., Walla et al., 2001) and for tasks involving phonology and syntax (Pugh et al., 1996; Jaeger et al., 1998). However, in general the existence of gender differences for language remains controversial, as considerable research has shown an absence of gender differences in both performance (e.g., Baxter et al., 2003; Clements et al., 2006) and neuroanatomical measures (e.g., Frost et al., 1999; Hund-Georgiadis et al., 2002; Sommer et al., 2004). Studies on age-related effects on visual speech have almost exclusively focused on differences between young and older adults (e.g., Sommers et al., 2005; Tye-Murray et al., 2007; Winneke and Phillips, 2011), and generally show that whereas older adults are poorer speech-readers than young adults, no age-related differences are reported for AV benefit, that is, use of visual speech to supplement auditory cues in AV speech perception. The few studies that have assessed the interaction of gender and age on visual speech show conflicting results (e.g., Dancer et al., 1994; Tye-Murray et al., 2007), possibly related to the age groups that have been compared. Older adults’ use of visual speech cues is likely sensitive to cognitive (e.g., Luchies et al., 2002; Der and Deary, 2006) and sensory (e.g., Davis, 1990) decline, such that the effect of AV-experience, and the interaction between AV-experience and gender may differ substantially for adults with normal sensory and cognitive abilities. In addition, research suggests ambiguity and a lack of sensitivity in the measurements typically used to quantify the visual contribution to AV speech perception (e.g., Ross et al., 2007; Winneke and Phillips, 2011). Consequently, to assess the interaction between age and gender, the current study measures the influence of gender on the use of visual speech cues in young and middle-aged adults prior to considerable sensory and cognitive decline, using alternative measurements of visual contribution.

Speech-reading may be narrowly defined as the ability to recognize different speech sounds based on visual cues from lip and facial movements. In general, previous research suggests that females are better at speech-reading than males, a difference which has been attributed to females being more active gazers than males (e.g., Berndl et al., 1986; Johnson et al., 1988). However, apart from this general trend, findings have been somewhat inconsistent, particularly related to which speech segments elicit a gender difference in speech-reading. Dancer et al. (1994) and Watson et al. (1996) showed that young adult females were better speech-readers than young adult males for words, but not for sentences. Strelnikov et al. (2009) also found that young adult females were better speech-readers for words but not for phonemes embedded in meaningless vowel-consonant-vowel disyllables. Contrarily, Johnson et al. (1988) found that females were better speech-readers than males for the consonants /b, p, t, d, k, g, n, m, v, and l/ when pronounced in a consonant-vowel context with the vowel /a/.

Research has indicated that the ability to identify visual speech (i.e., speech-reading) and the influence of visual cues on AV perception should be differentiated (e.g., Sommers et al., 2005; Irwin et al., 2006). While Irwin et al. (2006) did not find gender differences in speech-reading, they showed that females are more influenced by visual speech than males when an auditory syllable (/ba/) is accompanied by a brief visual syllable (99 ms /va/ or /da/–/tha/). Since no gender differences were found for full (660 ms) AV stimuli, they suggested that females’ more bilateral language processing generates more efficient AV speech processing, with observable gender differences emerging as task difficulty is increased by reducing the time window for binding visual and auditory information. That task difficulty influences the probability of observing gender differences in behavioral measurements of language has also been indicated elsewhere (e.g., Jaeger et al., 1998) and suggests that task difficulty should be varied in designs aimed to address gender differences in language. Despite the prevalence of including both male and female participants in studies of AV speech perception, few have directly addressed possible gender differences. Those studies which have tested gender differences have been consistent with Irwin et al. (2006), indicating that females are more influenced by visual cues than males in AV speech perception (Aloufy et al., 1996; Öhrström and Traunmüller, 2004). Öhrström and Traunmüller (2004) showed that females are significantly more influenced by the visual modality than males in perceiving AV incongruent Swedish vowels embedded in a syllable. Aloufy et al. (1996) tested English and Hebrew-speaking participants’ perception of AV incongruent consonants. In the English-speaking group females relied significantly less on auditory input than males. Females also showed a tendency for a visual bias although this difference was not statistically significant.

Differences in AV speech perception have also been seen across age groups. Sekiyama et al. (2014) calibrated signal-to-noise ratios (SNR) to achieve similar audio-only accuracy between normal-hearing young (18–21 years) and older adults (60–65 years). They found that, despite similar accuracy performance in the audio-only (AO) and visual-only (VO) conditions, the older adults gave more McGurk responses than the young adults, especially in high SNR conditions. However, response times by the older adults were longer than by the young adults in conditions including auditory stimuli, but not in the VO condition. The authors suggested that visual precedence due to delayed auditory processing may contribute to an enhanced visual influence, which may be accentuated by the additional processing strain caused by low SNRs. Although typically not revealing the age-related increase in AV integration found by Sekiyama et al. (2014), several studies have indicated that contrary to unimodal speech perception, AV integration is relatively unaffected in old adulthood (Sommers et al., 2005; Winneke and Phillips, 2011). Sommers et al. (2005) tested younger (18–24 years) and older adults’ (over 65 years) perception of auditory, visual, and AV words and sentences, with age-related differences in hearing acuity equalized using different intensities of babble noise. Whereas older adults generally demonstrated poorer speech-reading skills than young adults, no age-related differences were observed for AV benefit. These behavioral findings were replicated by Winneke and Phillips (2011) testing 17 younger (*M* = 24.5 years) and 17 older adults (*M* = 68.5 years), where the participants were primarily females (24 out of 34). Interestingly, ERP data revealed that older adults had a more pronounced facilitation of neural responses on AV speech trials than younger adults, interpreted as older adults being more able to benefit from visual speech cues in AV speech perception (Winneke and Phillips, 2011). The possibility arises that equal AV benefit scores may be caused by older adults’ deteriorating sensory and cognitive abilities being counterbalanced by attaining more proficient AV integration skills. As cognitive processing speed (Luchies et al., 2002; Der and Deary, 2006) and hearing acuity (Davis, 1990) show the most prominent decline after 60 years of age, new insights into the effect of AV-experience might be obtained by comparing the AV speech perception of young and middle-aged adults (less than 60 years; e.g., Alm and Behne, 2013). AV speech perception may be enhanced by increasing age-related AV experience before being counteracted by cognitive and sensory decline.

In addition to a simple increase in the amount of AV experience, development from young to middle adulthood may qualitatively modify the manner by which the cognitive and perceptual resources are used in AV speech perception. Even in normal-hearing adults, the small reduction in hearing acuity typically seen between young and middle adulthood may induce an experience-related modification in AV speech perception. Contextual noise is common in every day speech environments and the influence of such noise on speech perception may vary in the course of a lifespan. Recent findings indicate differences in speech reception thresholds between normal hearing young adults (19–26 years) and normal hearing middle-aged adult (51–63 years) for competing speech, music and steady noise maskers (Baskent et al., 2014). Although the participants were assessed as normal-hearing and had similar speech perception in quiet, small differences in audiometric thresholds were inferred to have resulted in the age-related differences in speech reception thresholds in the other background conditions. Such age dependent variations in the influence of noise may alter the way available cognitive resources are utilized in AV speech perception, for example, changing the relative processing of auditory and visual speech cues (e.g., Alm and Behne, 2014). Recent research also indicates that similar mechanism may be present for vision. Huyse et al. (2014) tested young (*M* = 20.9 years) and older adults (*M* = 68.3 years) with normal or corrected-to-normal vision. Whereas the young and older adults had similar scores on speech-reading and AV benefit for clear speech, older adults had significantly lower scores on both measurements for visually degraded speech. Whether these age-related effects are present already in middle-adulthood is not known, but, similar to auditory speech perception in noise (Baskent et al., 2014), these findings may imply that even for individuals with normal vision, small age-related changes in visual acuity may be an incentive for change in visual perception in adverse visual conditions, for example in situations where glasses or contact lenses are not used. Collectively these findings suggest that a sensitive relationship between AV experience and sensory acuity may gradually shift the contribution of the auditory and the visual signal in AV speech perception, and that these changes appear prior to significant sensory decline.

To the authors’ knowledge no studies have directly investigated the interaction of age and gender on AV speech perception between young and middle adulthood. An influence of age on gender differences would presuppose some experience-dependent flexibility in AV speech perception and research has suggested that both biological (Kulynych et al., 1994; Foundas et al., 2002) and environmental factors (e.g., Strelnikov et al., 2009) influence gender differences. Investigations of the origin of gender differences in mental rotation, the cognitive skill for which gender differences have been most consistently found (e.g., Linn and Petersen, 1985; Voyer et al., 1995), indicate that whereas hormones may contribute to development of gender differences (McGee, 1979; Kimura, 1992), brief training sessions have proven effective in leveling gender performance, with the effect still present when participants were retested 3 weeks later (Kass et al., 1998). Likewise for AV speech perception; whereas gender differences in the symmetry of brain regions involved in speech may contribute to gender differences in AV speech perception (Kulynych et al., 1994; Foundas et al., 2002), AV experience has been found to level gender differences in AV performance (e.g., Strelnikov et al., 2009). Research has shown that decline in auditory speech comprehension by the profoundly deaf is mitigated by acquisition of better speech-reading skills (Summerfield, 1992; Tyler et al., 1997; Grant et al., 1998) and this proficiency in speech-reading is maintained several years after cochlear implantation (Rouger et al., 2007). Strelnikov et al. (2009) found large gender differences in normal hearing adults for visual word recognition, whereas no such gender differences were found for experienced cochlear implanted patients. It appears that when adapting to reduce hearing acuity, males’ speech-reading skills improved over time to nearly equal that of females, which lead Strelnikov et al. (2009) to propose that gender differences in the utilization of visual speech cues may be due to differences in perceptual strategies that are sensitive to AV-experience.

Generally the behavioral research on AV speech perception reports relatively few and inconsistent findings on age and gender differences in the contribution of visual speech, especially for gender. Although gender differences in speech-reading are quite frequently reported (e.g., Johnson et al., 1988; Dancer et al., 1994; Watson et al., 1996; Strelnikov et al., 2009), few studies indicate that such gender differences in speech-reading affect AV speech perception. However, research typically assesses the contribution of visual speech on AV speech perception comparing differences in the amount of correct responses between AO stimuli and AV congruent stimuli in different noise conditions (i.e., AV benefit) or through changes in the amount of AV fusion responses to McGurk stimuli (McGurk and MacDonald, 1976). Arguably, what is assessed using these measurements is the ability to integrate the auditory and visual information, making the individual contribution of the auditory and visual modalities very hard to discern. For example, the balance of visual saliency and auditory saliency for optimal AV-integration responses to McGurk stimuli is not straightforward. Ross et al. (2007) found that AV-fusion is most likely to occur at intermediate SNRs, whereas extremely positive or negative SNRs favor the auditory or visual modality respectively. Analogously, it is difficult to say whether greater reliance on visual cues would result in more AV integration responses or less AV integration responses. Further, research has shown that age-related differences in brain activation patterns to AV speech are not reflected in age-related differences in behavioral measurements of AV benefit in AV speech perception (e.g., Winneke and Phillips, 2011), indicating that AV benefit may not be a particularly sensitive measurement of the contribution of visual speech on AV speech perception. Consequently, in our opinion, a measurement is needed that does not entail AV integration for evaluating the individual influence of the auditory and visual cues on AV speech perception. One approach would be to use AV incongruent stimuli and evaluate the amount of responses corresponding to the auditory input and the visual input individually. Such forced choice responses would be more independent of AV integration and reflect the reliance on or influence of the individual modalities more clearly.

The current study explores the interaction of age and gender using two measurements of visual contribution to AV speech perception: AV benefit and visual influence. AV benefit is calculated as the difference between correct responses in the AV congruent condition and in the corresponding AO condition, whereas visual influence is calculated as the difference between correct responses in the AO condition and the auditory responses in the AV incongruent condition (e.g., Sekiyama et al., 2003; Chen and Hazan, 2009). Based on these operationalizations, AV benefit may reflect the ability to correctly encode and integrate visual speech cues to predict or complement auditory cues in AV perception, resulting in enhanced speech identification compared to unimodal perception. Contrastingly, visual influence may reflect the inclination to rely on visual input in AV speech perception, and may reveal a more general AV perceptual strategy. In contrast to AV benefit, increased visual influence does not explicitly imply proficiency in AV integration (e.g., Irwin et al., 2006), but rather measures the relative dominance of one modality over the other in AV speech perception. Consequently, compared to AV benefit, visual influence may be a more sensitive measurement of the direct visual contribution on AV speech perception since it differentiates between audiovisual integration responses and visual responses. The current hypothesis is that, compared to males, females’ proficiency in speech-reading in young adulthood gives a basis for females to have a more dominant use of visual cues in AV speech perception in middle adulthood, and although such gender differences may not be evident for AV benefit, they are more likely to be observed with the more sensitive measure visual influence.

## Materials and Methods

### Design

A mixed repeated measures design was used to assess speech-reading, AV benefit and visual influence by young and middle-aged males and females using stimuli consisting of AO, VO, AV congruent and AV incongruent stop-vowel syllables produced by eight different talkers, varying in stop place of articulation (POA) and noise type.

### Participants

Forty Norwegian native speakers were recruited at the Norwegian University of Science and Technology (NTNU), including 10 young males (*M* = 25 years, SD = 2 years), 10 young females (*M* = 23 years, SD = 3 years), 10 middle-aged males (*M* = 53 years, SD = 3 years), and 10 middle-aged females (*M* = 55 years, SD = 3 years). The study was registered by the Norwegian Social Science Data Services, and all participants gave written consent prior to the experiment. Participants were all highly educated and naive to AV speech perception experiments. Prior to the experiment, hearing was assessed using a standard pure tone audiometry procedure (British Society of Audiology, 2004) and only those with hearing threshold levels below 20 dB for the frequencies 125, 250, 500, 1000, 2000, and 4000 Hz participated in the experiment. Four middle-aged males and one middle-aged female did not meet these criteria and did not continue on to the perception experiment. The average hearing threshold (dB HL) for young males (*M* = 2, SD = 2) did not significantly differ from young females [*M* = 3, SD = 3; *F*(1,19) = 1.54, n.s.], nor did middle-aged males (*M* = 7, SD = 3) from middle-aged females [*M* = 8, SD = 3; *F*(1,19) = 0.93, n.s.]. Vision was assessed with a self-report questionnaire and those participants who reported reduced vision wore prescription glasses or contact lenses during the experiment.

### Stimuli

The current study attempts to replicate the gender differences in speech-reading observed by Johnson et al. (1988) and therefore uses a selection of the same stop consonants and the same vowel context. **Table 1** shows the set of AO, VO, and AV stimuli that were created from audio and visual recordings of the four different syllables that differed in POA and voicing: labial /ba/ and /pa/, and velar /ga/ and /ka/. As shown in **Table 1**, congruent stimuli refer to stimuli in which the audio and visual components match for POA and incongruent stimuli refer to stimuli in which the audio and visual components differ in POA. Incongruent stimuli had two different stimulus structures: A_labial_V_velar_ and A_velar_V_labial_. All stimuli had congruent voicing. The AO, AV congruent and AV incongruent stimuli were all presented in quiet, 0 dB SNR babble and 0 dB SNR white noise, whereas VO stimuli were only presented in quiet.

**Table 1:** Stimuli used in the experiment.

Audio-only	Visual-only	AV-congruent		AV-incongruent	
		Audio	Video	Audio	Video
ba	ba	ba	ba	ba	ga^∗^
pa	pa	pa	pa	pa	ka^∗^
ga	ga	ga	ga	ga	ba^∗∗^
ka	ka	ka	ka	ka	pa^∗∗^

*The stimulus set used in the experiment included audio-only (AO), visual-only (VO), AV congruent and AV incongruent stimuli.*

*The AO, AV congruent and AV incongruent stimuli were presented in quiet, 0 dB SNR babble, and 0 dB SNR white noise. The same stimulus set was created for productions from eight talkers. ^∗^A_*labial*_V_*velar*._^∗∗^A_*velar*_V_*labial*_.*

#### Audio-Visual Recordings

Research suggests that AV task difficulty influences the probability of observing gender differences in AV speech perception (e.g., Irwin et al., 2006) and the current study therefore employed different noise backgrounds and different talkers to provide variability in AV task difficulty. Although considerable research has shown substantial differences in talker intelligibility influenced, for instance, by gender of the talker (Markham and Hazan, 2004), articulatory precision and fundamental frequency (Bradlow et al., 1996), consonantal contrast cues and vowel duration (Bond and Moore, 1994), most studies on the visual influence on AV speech perception have used only one talker (notable exceptions are Sekiyama and Tohkura, 1993; Traunmüller and Öhrström, 2007; Chen and Hazan, 2009). Consequently, AV recordings of two young male talkers, two middle-aged male talkers, two young female talkers and two middle-aged female talkers were carried out in the Speech Laboratory at the Department of Psychology, NTNU. All talkers had an urban Eastern-Norwegian dialect to which most Norwegians are accustomed. The male talkers were clean-shaven. Prior to the recordings artificial distractors, such as glasses and jewelery, were removed.

The talkers were told to maintain a relatively flat intonation, to avoid any pronounced rise or fall in pitch toward the end of syllables. They were also instructed to minimize facial movement irrelevant to speech, such as eye blinks. To avoid any visual distractions in the stimuli, the talkers were seated in front of a featureless gray wall.

The AV recordings were conducted in a sound-insulated studio where each talker sat facing a SANYO VPC-FH1 camera at a 90 cm distance. A Røde NT1-A microphone was positioned 50 cm to the left and 10 cm above the head of the talkers to be out of line from the camera. Two parallel audio recordings were made: one from the video camera’s internal microphone and one from an external microphone (i.e., the Røde NT1-A). The sound from the external microphone went via a RME FIREFACE 400 soundcard to an Apple Macintosh G5 computer, where Praat version 5. 1 (Boersma and Weenink, 2009) was used to record two audio channels at a 48 kHz sampling rate.

The four consonant-vowel syllables employed in the study contained the stop consonants /b, p, g/ and /k/ succeeded by the vowel /a/ (**Table 1**). Each syllable was repeated eight times. The video file was segmented into separate syllables, using the software MPEG Streamclip 1.9.2 and the audio files from the external microphone were segmented with Praat version 5.1 (Boersma and Weenink, 2009). The segmented MPEG-4 video clips had a rate of 30 frames per second and a 1920 × 1080 pixel resolution.

The segmented video and audio files were independently rated by three different evaluators. Highly rated video segments were those in which syllable articulations were explicit and eye blinks or other unwanted facial gestures few. A highly rated audio segment implied a natural syllable pronunciation and a relatively even intonation, accompanied by no unwanted noise, such as that from movement in the recording environment. For each of the eight talkers, two recordings of each of the four syllables (total of 64 syllables) were selected based on the highest additive audio and visual ratings (see Alm and Behne, 2013 for details about the rating).

All audio syllable segments were adjusted to the same unweighted sound pressure level in Praat. The average length of the auditory syllables was 400 ms (*range* = 272–537 ms) measured from the consonant release to the end of the vowel.

#### Assembling Audio-Visual Stimuli

As shown in **Table 1**, four congruent and four incongruent AV stimuli were used in the experiment. To create an AV congruent stimulus the audio syllable from the external microphone (i.e., Røde NT1-A) was first synchronized with the same syllable from the camera microphone in Logic Pro 8.0.2. Then the video clip’s original auditory syllable recording was replaced by the corresponding syllable from the external microphone in AVID Media Composer. The incongruent stimuli were produced in the same manner; except that the video clip’s original auditory syllables were substituted with external auditory syllables that differed in POA. The video clips were cut to a total length of 1520 ms, ensuring that the consonant release of all syllables was initiated during the 16th frame (between 640 and 680 ms). The resulting congruent and incongruent AV syllables constituted the quiet condition of the experiment.

#### Noise Signal

The study employed two types of auditory maskers: babble and white noise. Whereas babble noise occurs more often in natural environments (cf. e.g., Alm et al., 2009), in laboratory studies on AV speech, white noise is more commonly used as a masker (e.g., Dodd, 1977; Easton and Basala, 1982; Fixmer and Hawkins, 1998). Research suggests the effects of masking speech with babble or white noise may be different for phonetic attributes such as for POA and voicing (Alm et al., 2009).

The babble noise was recorded during lunchtime in a cafeteria at NTNU, using an Okay II DM-801 microphone connected to a SHG Note 40750 laptop via its built-in soundcard, and using a sampling frequency of 48 kHz. A segment of the recording was extracted in which babble was prominent and other sounds, such as coughs and the rattling of cutlery, were minimal. Individual voices could not be differentiated in the babble segment. The white Gaussian noise was generated using the “create sound” function in Praat (Boersma and Weenink, 2009). The babble and the white noise segments were cut to a length of 1520 ms, equalling the length of the video clips. The noise segments were then adjusted to the same unweighted sound pressure level as the syllables using Praat (Boersma and Weenink, 2009).

That the two noise segments had the same length as the video clips enabled initiation of the noise signals 640–680 ms prior to the auditory speech signals and prevented perceptual artifacts caused by a sudden onset of noise. The noise segments were added to the AO, AV congruent and AV incongruent stimuli in AVID Media Composer and resulted in stimuli with three different audio backgrounds: 0 dB SNR white and babble noise, and quiet. The VO stimuli were only presented in quiet.

### Procedure

Participants were seated facing monitors (1920 × 1200 pixels) at ∼70 cm distance, wearing AKG K271 stereo closed dynamic circumaural studio headphones. The sound level was fixed at 68 dBA (corresponding to a frontally incident free-field sound pressure level around 68 dBA).

Participants were presented six stimulus blocks: two repetitions of an AV block (AV-congruent and AV-incongruent intermixed), two repetitions of an AO block, and two repetitions of a VO block (see **Table 1**). Each block contained two productions of each syllable by each of the eight talkers. With three audio backgrounds each AV-stimulus block contained 384 stimuli and each AO stimulus block contained 192 stimuli. The VO stimulus blocks contained 64 stimuli. Stimuli were independently randomized for each repetition.

For each trial the participant’s task was to watch and listen to the syllable and press a button on a Cedrus RB-730 seven-button response pad to indicate which among six alternative syllables (ba, da, ga, ka, pa, and ta) best corresponded to the syllable perceived. Because of possible ambiguity with incongruent AV signals, the participants were told that no answer was wrong. To ensure that the participants received both auditory and visual input, frequent between-trial reminders instructed the participants to look at the talker’s face throughout the duration of each clip. The experiment took ∼1 h.

## Results

### Audio-Only, Visual-Only and AV-Congruent Control Conditions

Audio-only data were analysed with a repeated measures ANOVA where within subject variables were background (quiet, babble and white noise) and stop consonant (/ba/, /ga/, /pa/ and /ka/), between subject variables were age and gender and the dependent variable was percentage correct responses. A correct response required a perfect match, signifying that the response corresponded to the stimulus in both POA and voicing. As shown in **Table 2**, in the AO condition no significant age [*F*(1,36) = 1.14, *p* = 0.29, η^2^ = 0.03, power = 0.18] or gender [*F*(1,36) = 0.18, *p* = 0.67, η^2^ = 0.005, power = 0.07] differences were found. Overall in the AO condition, the percentage of correct responses by young males (*M* = 75%, SE = 2) and females (*M* = 76%, SE = 2) was similar to middle-aged males (*M* = 78%, SE = 2) and females (*M* = 78%, SE = 2). As expected a main effect was found for stop consonant [*F*(3,108) = 109.48, *p* < 0.001; e.g., Miller and Nicely, 1955] and background [*F*(2,72) = 535.62, *p* < 0.001; e.g., Parikh and Loizou, 2005]. As can be observed in **Table 2**, labials, especially the syllable /pa/, received considerably lower identification scores in babble and white noise than velars. This finding is supported by Parikh and Loizou (2005) who found that in -5 db SNR babble contexts labial consonants embedded in vowel-consonant-vowel disyllables received lower identification scores than velar consonants. They speculated that as babble masks lower frequencies more than high frequencies, labials, with equal spread of energy across frequencies or spectral prominence in low frequencies would be more affected than velars with mid-frequency prominence. That similar effects are observed for the flat spectrum white noise also fit well with such an explanation. Importantly, for the current assessment, neither background nor stop consonant interacted significantly with age or gender.

**Table 2:** Syllable identification scores.

	Background	Syllable	A-only				V-only		AV congruent		Incongruent AV stimuli	AV incongruent			
			Perfect match		POA match		POA match					Audio POA match		AV fusion/error	
			Mean %	SE	Mean %	SE	Mean %	SE	Mean %	SE		Mean %	SE	Mean %	SE
Young male	Quiet	BA	93	2	96	2	92	2	97	1	BAGA	55	9	40	10
		GA	92	1	98	1	63	6	97	2	GABA	94	2	3	1
		PA	87	3	88	3	89	2	94	1	PAKA	57	9	32	9
		KA	93	1	93	1	52	6	98	1	KAPA	95	3	3	0
	Babble	BA	71	4	75	4	–	–	87	2	BAGA	27	6	36	7
		GA	82	2	88	1	–	–	93	1	GABA	80	4	8	1
		PA	55	6	60	6	–	–	84	2	PAKA	21	5	32	6
		KA	90	2	93	1	–	–	93	2	KAPA	60	4	1	1
	White	BA	51	4	58	5	–	–	77	3	BAGA	16	4	41	7
		GA	78	3	87	3	–	–	80	2	GABA	63	5	13	2
		PA	35	6	38	7	–	–	91	2	PAKA	14	4	25	7
		KA	73	4	75	4	–	–	85	3	KAPA	32	3	5	1
Young female	Quiet	BA	93	2	94	2	96	2	95	1	BAGA	53	9	35	10
		GA	97	1	99	1	81	6	96	2	GABA	98	2	2	1
		PA	92	3	92	3	96	2	97	1	PAKA	62	9	26	9
		KA	93	1	93	1	68	6	99	1	KAPA	94	3	2	0
	Babble	BA	71	4	76	4	–	–	86	2	BAGA	31	6	16	7
		GA	86	2	91	1	–	–	92	1	GABA	84	4	7	1
		PA	52	6	57	6	–	–	89	2	PAKA	24	5	20	6
		KA	93	2	94	1	–	–	95	2	KAPA	57	4	1	1
	White	BA	46	4	53	5	–	–	79	3	BAGA	18	4	21	7
		GA	81	3	92	3	–	–	84	2	GABA	63	5	6	2
		PA	35	6	39	7	–	–	93	2	PAKA	10	4	24	7
		KA	78	4	80	4	–	–	85	3	KAPA	27	3	3	1
Middle-aged male	Quiet	BA	96	2	98	2	91	2	97	1	BAGA	66	9	30	10
		GA	97	1	100	1	66	6	96	2	GABA	96	2	4	1
		PA	95	3	96	3	91	2	98	1	PAKA	72	9	21	9
		KA	93	1	93	1	52	6	99	1	KAPA	93	3	0	0
	Babble	BA	70	4	78	4	–	–	81	2	BAGA	46	6	20	7
		GA	84	2	90	1	–	–	93	1	GABA	80	4	10	1
		PA	49	6	54	6	–	–	88	2	PAKA	32	5	23	6
		KA	96	2	96	1	–	–	96	2	KAPA	62	4	0	1
	White	BA	54	4	62	5	–	–	78	3	BAGA	31	4	19	7
		GA	80	3	89	3	–	–	81	2	GABA	56	5	6	2
		PA	39	6	43	7	–	–	88	2	PAKA	17	4	23	7
		KA	78	4	79	4	–	–	87	3	KAPA	31	3	4	1
Middle-aged female	Quiet	BA	97	2	98	2	98	2	96	1	BAGA	51	9	43	10
		GA	97	1	98	1	64	6	95	2	GABA	86	2	5	1
		PA	96	3	97	3	97	2	98	1	PAKA	55	9	35	9
		KA	93	1	93	1	57	6	99	1	KAPA	81	3	0	0
	Babble	BA	76	4	79	4	–	–	86	2	BAGA	27	6	27	7
		GA	84	2	89	1	–	–	90	1	GABA	64	4	8	1
		PA	48	6	53	6	–	–	87	2	PAKA	25	5	26	6
		KA	95	2	97	1	–	–	93	2	KAPA	38	4	0	1
	White	BA	60	4	68	5	–	–	83	3	BAGA	20	4	23	7
		GA	83	3	92	3	–	–	83	2	GABA	37	5	8	2
		PA	31	6	36	7	–	–	93	2	PAKA	14	4	23	7
		KA	77	4	79	4	–	–	86	3	KAPA	13	3	2	1

*Young and middle-aged males and females’ mean percentage perfect match responses in the AO and AV congruent conditions, mean percentage POA match responses in the AO and VO condition, and mean percentage auditory POA match responses and AV fusion/error responses for the AV incongruent conditions. The stimuli varied in voicing (voiced and voiceless), POA (labial and velar consonants), and auditory background (quiet, babble and white noise).*

Visual-only data were analyzed with a repeated measures ANOVA where the within subject variable was stop consonant (/ba/, /ga/, /pa/ and /ka/), between subject variables were age and gender and the dependent variable was percentage correct POA responses regardless of voicing, since visual discrimination is difficult for consonants belonging to the same viseme class (Kent, 1997). As illustrated in **Table 2**, a significant main effect was obtained for gender [*F*(1,36) = 5.72, *p* = 0.022, η^2^ = 0.13, power = 0.74], where females (*M* = 82%, SE = 2) had significantly more correct POA responses than males (*M* = 75%, SE = 2). No significant effect was obtained for age [*F*(1,36) = 0.85, *p* = 0.36, η^2^ = 0.02, power = 0.15] or interaction between age and gender [*F*(1,36) = 1.27, *p* = 0.27]. As expected a significant effect of stop consonant was obtained [*F*(3,108) = 94.59, *p* < 0.001], and in line with previous research, labials resulted in more correct responses than velars (e.g., Walden et al., 1977; Benguerel and Pichora-Fuller, 1982). Stop consonant did not significantly interact with age or gender.

Audio-visual congruent data were analyzed with a repeated measures ANOVA where within subject variables were background (quiet, babble and white noise) and stop consonant (/ba/, /ga/, /pa/ and /ka/), between subject variables were age and gender and the dependent variable was percentage correct responses. As shown in **Table 2**, no significant age [*F*(1,36) = 0.04, n.s] or gender [*F*(1,36) = 0.98, n.s] differences were obtained for AV congruent stimuli. Overall, the percentage of correct responses for young males (*M* = 90%, SE = 1) and females (*M* = 91%, SE = 1) was almost the same as for middle-aged males (*M* = 90%, SE = 1) and females (*M* = 91%, SE = 1). Main effects were found for stop consonant [*F*(3,108) = 16.94, *p* < 0.001] and background [*F*(2,72) = 190.06, *p* < 0.001], but neither interacted significantly with age or gender.

The high percentage of correct responses for the AO stimuli in the quiet condition implies that the auditory stimuli are good tokens of their respective categories. The percentage of correct responses in the AO condition declines sharply as noise is introduced, but with the visual cues offered in the AV-congruent condition, participants have near perfect responses in noise. Along with the high percentage correct in the VO condition, this indicates that the visual stimuli are good tokens of their respective categories. Furthermore, the high percentage of correct responses found for the AV congruent stimuli makes it unlikely that differences for the AV incongruent stimuli are due to chance responses.

Visual-only data were analyzed with a repeated measures ANOVA where the within subject variable was stop consonant (/ba/, /ga/, /pa/ and /ka/), between subject variables were age and gender and the dependent variable was percentage correct POA responses regardless of voicing, since visual discrimination is difficult for consonants belonging to the same viseme class (Kent, 1997). As illustrated in **Table 2**, a significant main effect was obtained for gender [*F*(1,36) = 5.72, *p* = 0.022, η^2^ = 0.13, power = 0.74], where females (*M* = 82%, SE = 2) had significantly more correct POA responses than males (*M* = 75%, SE = 2). No significant effect was obtained for age [*F*(1,36) = 0.85, *p* = 0.36, η^2^ = 0.02, power = 0.15] or interaction between age and gender [*F*(1,36) = 1.27, *p* = 0.27]. As expected a significant effect of stop consonant was obtained [*F*(3,108) = 94.59, *p* < 0.001], and in line with previous research, labials resulted in more correct responses than velars (e.g., Walden et al., 1977; Benguerel and Pichora-Fuller, 1982). Stop consonant did not significantly interact with age or gender.

Audio-visual congruent data were analyzed with a repeated measures ANOVA where within subject variables were background (quiet, babble and white noise) and stop consonant (/ba/, /ga/, /pa/ and /ka/), between subject variables were age and gender and the dependent variable was percentage correct responses. As shown in **Table 2**, no significant age [*F*(1,36) = 0.04, n.s] or gender [*F*(1,36) = 0.98, n.s] differences were obtained for AV congruent stimuli. Overall, the percentage of correct responses for young males (*M* = 90%, SE = 1) and females (*M* = 91%, SE = 1) was almost the same as for middle-aged males (*M* = 90%, SE = 1) and females (*M* = 91%, SE = 1). Main effects were found for stop consonant [*F*(3,108) = 16.94, *p* < 0.001] and background [*F*(2,72) = 190.06, *p* < 0.001], but neither interacted significantly with age or gender.

The high percentage of correct responses for the AO stimuli in the quiet condition implies that the auditory stimuli are good tokens of their respective categories. The percentage of correct responses in the AO condition declines sharply as noise is introduced, but with the visual cues offered in the AV-congruent condition, participants have near perfect responses in noise. Along with the high percentage correct in the VO condition, this indicates that the visual stimuli are good tokens of their respective categories. Furthermore, the high percentage of correct responses found for the AV congruent stimuli makes it unlikely that differences for the AV incongruent stimuli are due to chance responses.

### Age and Gender Differences in AV Benefit

Audio-visual benefit implies the ability to correctly encode and integrate visual speech cues during AV speech perception, resulting in improved identification scores compared to unimodal identification scores (e.g., Sumby and Pollack, 1954; Erber, 1969; MacLeod and Summerfield, 1987). AV benefit, or the size of the positive visual effect, can be described by the difference between the response match in the AV congruent condition and the AO condition (e.g., Sekiyama et al., 2003; Chen and Hazan, 2009).

Audio-visual benefit was operationalized as the difference between percentage correct POA match responses for AV congruent stimuli and percentage correct POA match responses for AO stimuli with the corresponding auditory syllable (Sekiyama et al., 2003; Chen and Hazan, 2009). The data were analyzed with a repeated measure ANOVA where within subject variables were stop consonant and background, between subject variables were age and gender and the dependent variable was percent AV benefit for AV perception. No significant age [*F*(1,36) = 1.44, *p* = 0.24, η^2^ = 0.04, power = 0.22] or gender [*F*(1,36) = 0.01, *p* = 0.92, η^2^ < 0.001, power = 0.051] effects were obtained for AV benefit. Young males (*M* = 16, SE = 2) had similar AV benefit as young females (*M* = 16, SE = 2) and middle-aged males (*M* = 15, SE = 2) had similar AV benefit as middle-aged females (*M* = 15, SE = 2).

### Age and Gender Differences in Visual Influence

Visual influence denotes the degree to which a perceiver relies on input from the auditory and visual modalities in AV speech perception. Visual influence can be described by the difference between the auditory accuracy in the AO condition and the percent auditory responses in the AV incongruent condition (e.g., Sekiyama et al., 2003; Chen and Hazan, 2009). Contrary to AV benefit, the degree of visual influence may reflect differences in AV perceptual strategy (i.e., the degree of reliance on the visual input) and can, but does not explicitly require integration of visual and auditory signals.

**FIGURE 1:**
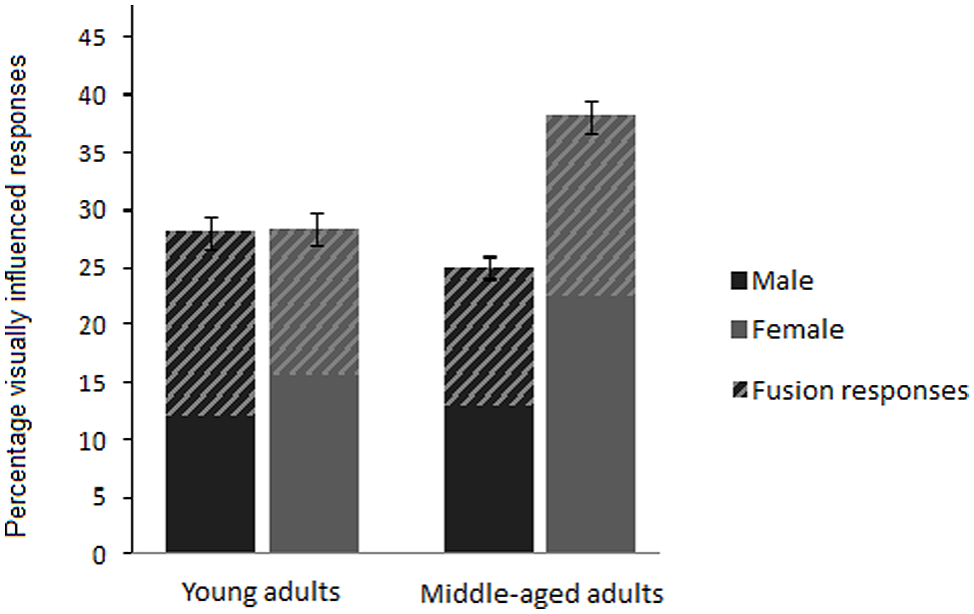
**Overall mean percentage visually influenced responses given by young and middle-aged males and females.** Visually influenced responses match the visual component for A_labial_V_velar_ and A_velar_V_labial_ stimuli (solid areas) and include fusion responses for A_labial_V_velar_ stimuli (hatched areas). Error bars for visually influenced responses show SE.

**FIGURE 2:**
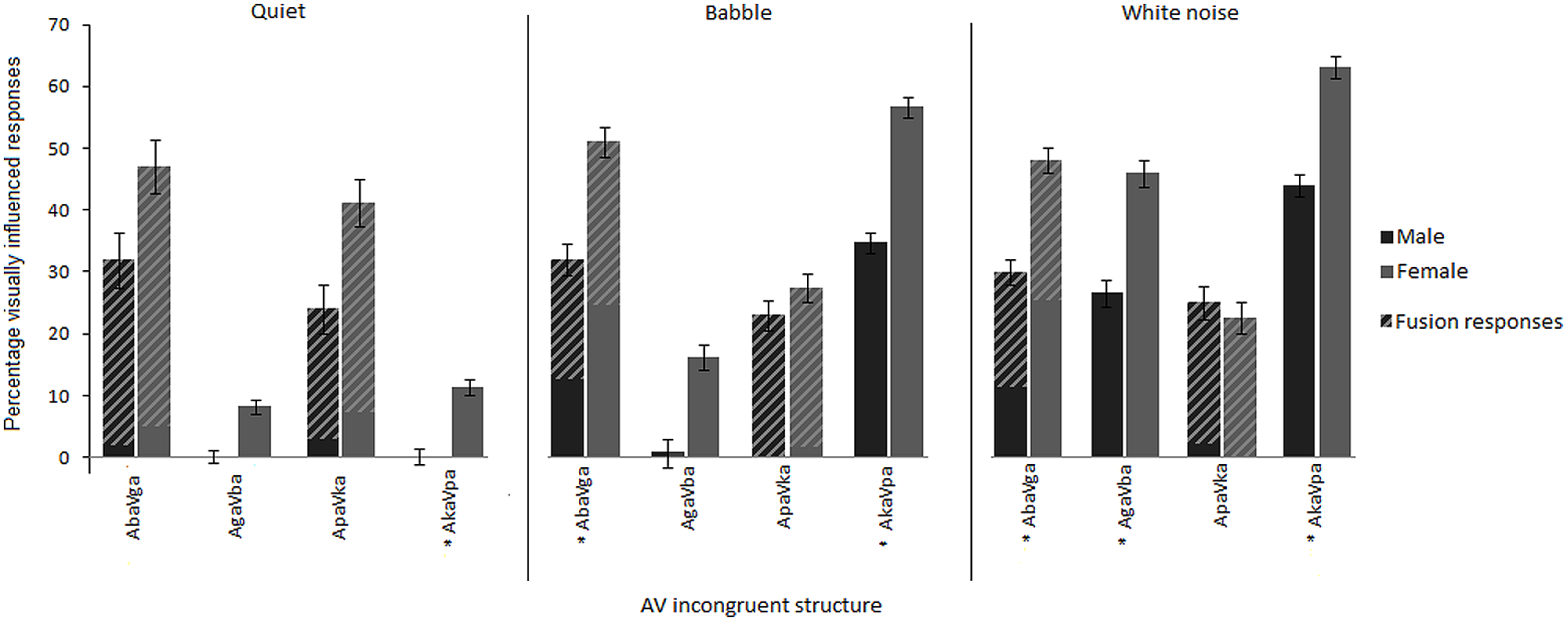
**Mean percentage visually influenced responses given by middle-aged males and females in quiet, babble and white noise for the different AV incongruent structures.** Visually influenced responses match the visual component for A_labial_V_velar_ and A_velar_V_labial_ stimuli (solid areas) and include fusion responses for A_labial_V_velar_ stimuli (hatched areas). Asterisks indicate significant (*p* < 0.05) gender differences. Error bars are given in SE.

**FIGURE 3:**
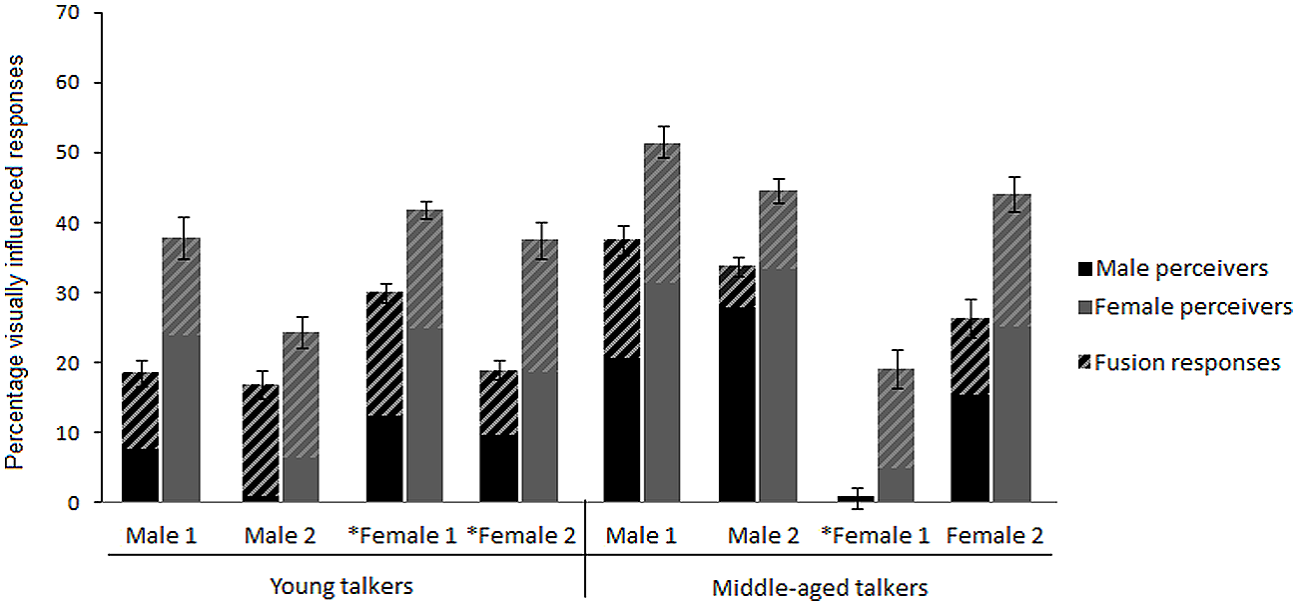
**Mean percentage visually influenced responses given by middle-aged males and females for talkers differing in gender and age.** Responses are collapsed across AV incongruent structure and auditory background. Asterisks indicate significant (*p* < 0.05) perceiver gender differences. Error bars are given in SE.

Visual influence was operationalized as the difference between percentage correct POA match responses for AO stimuli and percentage auditory POA match responses for AV incongruent stimuli with the corresponding auditory syllable (Sekiyama et al., 2003; Chen and Hazan, 2009). The AV incongruent stimuli consisted of voiced and voiceless AV syllable pairs, for which the auditory and visual components differed in stop consonant POA (see **Table 1**) and the participants, responded with the syllable alternatives /ba/, /da/, /ga/, /pa/, /ta/ and /ka/. Because members of the same viseme class are difficult to discern visually (Kent, 1997), responses that corresponded to the visual component of AV incongruent stimuli in POA but not voicing, were analyzed as visually influenced responses. In addition, in cases where incongruent A_labial_V_velar_ stimuli lead to audiovisual fusion responses (McGurk and MacDonald, 1976), fusion responses were interpreted as visually influenced responses based on findings, for example, that adding moderate auditory noise to AV incongruent stimuli leads to an increase in unambiguous visual responses as well as a shift toward more fusion responses (e.g., Dodd, 1977; Easton and Basala, 1982; Fixmer and Hawkins, 1998). For clarity, the portion of fusion responses is indicated in **Figures 1–3**, since, compared to responses matching the visual component, fusion responses represent a more equivocal measure of visual contribution to AV speech perception. Given that fusion responses for incongruent A_velar_V_labial_ stimuli are rare (McGurk and MacDonald, 1976), the few occurrences in the current study are treated as error and not included in the calculation of visual influence.

In summary, for the incongruent A_labial_V_velar_ stimuli Formula [1] was used to calculate visual influence. Consequently, for A_labial_V_velar_ stimuli /ba/ and /pa/ responses indicated auditory influence and /ga/, /ka/, /da/ and /ta/ indicate visual influence.

(1) $$\mathrm{POA}\mathrm{match}\mathrm{for}{\mathrm{AO}}_{\mathrm{labial}}-\mathrm{auditory}\mathrm{POA}\mathrm{match}{\mathrm{forA}}_{\mathrm{labial}}V_{\mathrm{velar}}$$

For the incongruent A_velar_V_labial_ stimuli Formula [2] was used to calculate visual influence. Consequently, for A_velar_V_labial_ stimuli /ga/ and /ka/ responses indicated auditory influence, /ba/ and /pa/ visual influence, and /da/ and /ta/ error. Overall the percentage of error responses was comparable for young males (*M* = 6, SD = 4), young females (*M* = 4, SD = 3), middle-aged males (*M* = 4, SD = 4) and middle-aged females (*M* = 4, SD = 4).

(2) $$\mathrm{POA}\mathrm{match}\mathrm{for}{\mathrm{AO}}_{\mathrm{velar}}-\mathrm{auditory}\mathrm{POA}\mathrm{match}\mathrm{for}A_{\mathrm{velar}}V_{\mathrm{labial}}-\mathrm{POA}\mathrm{error}\mathrm{responses}$$

The visual influence data calculated using Formula [1] and [2] were analyzed with two repeated measures ANOVAs and *p*-values from all *post hoc* analyses were collectively adjusted using Bonferroni–Holm corrections (Holm, 1979). A first repeated measures analysis was conducted to assess the interaction between age and gender for visual influence, and was based on the within subject variables AV incongruent structure (i.e., A_ba_V_ga_, A_pa_V_ka_, A_ga_V_ba_, and A_ka_V_pa_) and background, the between subject variables age and gender, with percent of visually influenced responses as the dependent variable. The analysis revealed significant main effects for AV incongruent structure [*F*(3,108) = 77.27, *p* < 0.001], background [*F*(2,72) = 33.80, *p* < 0.001], and gender [*F*(1,36) = 7.44, *p* = 0.01, η^2^ = 0.17, power = 0.76]. Although no significant main effect was found for age [*F*(1,36) = 1.71, *p* = 0.20, η^2^ = 0.05, power = 0.25], a significant interaction effect between age and gender was obtained [*F*(1,36) = 6.84, *p* = 0.013, η^2^ = 0.16, power = 0.72]. As **Figure 1** depicts, *post hoc* analyses of the interaction between age and gender revealed that middle-aged females had significantly more visually influenced responses than middle-aged males [*t*(18) = -4.18, *p* = 0.001, *r* = 0.70], young males [*t*(18) = -2.14, *p* = 0.05, *r* = 0.45], and young females [*t*(18) = -2.43, *p* = 0.05, *r* = 0.50]. Middle-aged males’ visual influence was similar to that of young males and young females, for which no significant gender differences were obtained. Because no gender differences were obtained for the young adults the following analyses focus on the middle-aged adults.

To assess the consistency of the gender effect for middle-aged adults, *post hoc* comparisons between middle-aged males and females for the different AV incongruent structures and different backgrounds were conducted. As **Figure 2** shows, with the exception of the visual influence of /ka/ (i.e., A_pa_V_ka_) in white noise, middle-aged females consistently had more visually influenced responses than middle-aged males for all AV incongruent structures and all backgrounds. Related to the effect of noise, the gender difference in visually influenced responses is comparable across backgrounds for the A_ba_V_ga_ stimuli, slightly more pronounced in noise for the A_ga_V_ba_ and A_ka_V_pa_ stimuli, and most pronounced in quiet for the A_pa_V_ka_ stimuli. Hence, although there is a tendency toward more significant gender effects in noise across the different AV incongruent structures, the notion that gender differences in AV speech perception would be more observable when the AV task difficulty is increased by auditory noise is generally not substantiated by the data.

To further investigate the resilience of the perceiver gender differences in visual influence, a second repeated measures analysis assessed middle-aged males and females’ percentage visually influenced responses in AV perception for the eight different talkers, collapsed across AV incongruent structure and background. Significant main effects were obtained for talker [*F*(7,12) = 21.72, *p* < 0.001] and perceiver gender [*F*(1,18) = 14.46, *p* = 0.005], and as **Figure 3** depicts, middle-aged females consistently gave more visually influenced responses than males for all talkers. *Post hoc* comparisons only revealed significant perceiver gender differences for female talkers. To follow up on this finding, talker intelligibility in the AO and VO conditions were analyzed, and in accordance with previous research (Bradlow et al., 1996; Markham and Hazan, 2004) results indicate that female talkers (*M* = 79%, SD = 6) were more auditorily intelligible than male talkers [*M* = 75%, SD = 6; *t*(40) = 5.96, *p* < 0.001], whereas no significant difference was obtained for visual intelligibility [*t*(40) = 0.61, n.s.]. Further, the middle-aged male and female perceivers had very similar auditory identification scores for both the male [*t*(18) = -0.07, n.s.] and the female talkers [*t*(18) = -0.15, n.s.]. Most importantly, results revealed the clear general pattern across the different talkers, with middle-aged females consistently giving more visually influenced responses compared to middle-aged males.

### Statistical Power and Sample Size

Effect size and statistical power have been provided for the significant and non-significant age and gender differences in AV benefit and visual influence, as well as for percent correct VO and AO responses. The observed statistical power indicates that the design and the sample size are adequate for detecting medium effects (0.09 < η^2^ < 0.25; Cohen, 1988), that is, effects of variables that explain more than nine percent of the variation in the dependent variable. All reported medium effects related to age and gender differences and their interactions exceeded 70% detection probability, and are generally quite close to the 80% detection probability recommended for the behavioral sciences (Cohen, 1988). However, as summarized in **Table 3**, the current study could lack statistical power for detection of small effects (0.01 < η^2^ < 0.09), that is, effects of variables that explain between 1 and 9 percent of the variation in the dependent variable. Since the study employed a relatively small sample size (*N* = 40), *post hoc* power analyses (Suresh and Chandrashekara, 2012) were conducted to investigate whether a reasonable increase in the sample size would benefit the detection rates considerably. The results in **Table 3** indicate that a sample size of ∼160 participants would be necessary to achieve an 80% probability for detecting all the small effects, and although practically achievable, whether the scientific importance of these small effects merits such a substantial increase in sample size is debatable. All the potential small effects are related to age differences and the means and the effect sizes indicate that age generally explains a very small portion of the variation in the current measurements. For example, age would only explain four percent of the variation in AV benefit, with a meager two percent difference in AV benefit between young and middle-aged adults. For AV benefit the effect size and the results of the power analysis therefore seem to be in agreement with previous research (e.g., Winneke and Phillips, 2011) which indicate that whereas an age-related difference may exist, AV benefit is not a particularly sensitive measurement of age-related differences in visual speech cues’ contribution to AV speech perception.

**Table 3:** Power analyses.

	Young adult	Middle-aged adults				Hypothetical sample size
	(Mean)	(Mean)	*p*	η^2^	Power %	for 80% power
Correct responses A-only	75,7	77,7	0.29	0.03	18	110
Correct responses V-only	79,7	76,9	0.36	0.02	15	160
Visual benefit	16,5	14,5	0.24	0.04	22	88
Visual influence	28	31,7	0.20	0.05	25	74

*List of small effects (0.01 < η^2^ < 0.09) that did not reach statistical significance (*p* > 0.05) in the current study.*

*The power of the current experiment to detect such small effects and the hypothetical sample size necessary to obtain recommended power of 80% is reported.*

### Summary of the Results

The results of the AO, VO, and AV control conditions revealed that the syllables used in the experiment were good tokens of their respective categories, and the results of the VO condition indicate that females are better speech-readers than males. Consistent with the hypothesis, the main analyses revealed a significant gender difference in visual influence for middle-aged adults, whereas no gender difference was observed for AV benefit. Middle-aged females gave more visually influenced responses than middle-aged males across stop consonants and talkers.

## Discussion

Research indicates that females are more proficient speech-readers than males (e.g., Johnson et al., 1988; Dancer et al., 1994; Watson et al., 1996; Strelnikov et al., 2009), but whether this proficiency interacts with age to produce gender differences in the use of visual cues in AV speech perception in middle adulthood has not been assessed. Previous research on age-related effects with AV speech perception has tended to compare young and older adults (e.g., Sommers et al., 2005; Tye-Murray et al., 2007; Winneke and Phillips, 2011), such that the sensory and cognitive decline associated with old age may have negated positive effects of age-related AV experience (e.g., Alm and Behne, 2013) and may hence provide an incomplete account of the interaction between age and gender in AV speech perception. In addition, the measurements typically used in AV speech perception research, such as McGurk fusion responses (McGurk and MacDonald, 1976) and AV benefit, may not be ideal for assessing the visual contribution to AV speech perception, particularly since the individual contribution of the auditory and visual modality are difficult to discern in measures focusing on AV integration. Consequently, as the sensitivity of the measures may influence the probability of exposing age and gender differences in visual contribution to AV speech perception, the current study used the measure visual influence to complement the arguably less sensitive measure AV benefit. The prediction was that, compared to males, females’ proficiency in speech-reading in young adulthood (20–30 years) would result in females showing greater reliance on visual cues in AV speech perception in middle adulthood (50–60 years), with gender differences more likely to emerge with visual influence than with the less sensitive measure AV benefit.

As predicted females had significantly more correct responses to VO stimuli than males for both young and middle-aged adults and these findings are consistent with the results obtained by Johnson et al. (1988) using similar stop consonants and vowel context. However, these gender differences in VO performance (i.e., speech-reading) did not contribute to significant gender differences in AV benefit. Although the potential benefit of visual cues was considerable in babble and white noise, for which AO identification of labials was difficult, increasing the task difficulty with auditory noise only lead to a negligible and non-significant gender difference in AV benefit. AV benefit likely depends on AV integration skills (e.g., Sommers et al., 2005) and previous research indicates that the ability to extract visual speech (i.e., speech-reading) and the ability to integrate auditory and visual speech may be different perceptual abilities (e.g., Grant and Seitz, 1998; Sommers et al., 2005). Thus, the lack of relationship between speech-reading and AV benefit for AV speech was not unexpected.

Contrary to the results for AV benefit, and consistent with the hypothesis, the results for visual influence revealed a clear effect of gender for middle-aged adults, whereas no gender effect was found for young adults. These findings highlight the importance of a lifespan perspective, with its intermediate phases, when assessing gender influences in AV speech perception. Taking into account the amount of AV speech literature and the prevalence of using young gender-balanced participant groups, the general lack of gender differences reported for AV speech perception in young adulthood may indicate that (1) gender differences in the use of visual cues in AV speech perception are negligible in young adulthood and/or (2) sufficiently sensitive measurements of visual contribution and sufficiently demanding tasks are necessary for observable gender differences to emerge. The current findings are more in line with the notion of negligible gender differences in the use of visual cues in AV speech perception in young adulthood, since inclusion of talker variability as well as babble and white noise did not lead to observable gender differences for either AV benefit or visual influence for young adults. These findings are inconsistent with the results by Öhrström and Traunmüller (2004) and Irwin et al. (2006). However, those studies tested participants varying substantially in age [18–49 years for Irwin et al. (2006) and 16–48 years for Öhrström and Traunmüller (2004)] and age was not treated as a factor in either. Notably, experiment two in Irwin et al. (2006) replicated the findings for the 18–49 year old group testing solely undergraduates (18–22 years). This replication with young adults indicates that gender differences may emerge in young adulthood for certain demanding AV speech perception tasks, possibly in particular when visual information is manipulated.

The most notable observation across different talkers and consonants is a clear general pattern showing that middle-aged female perceivers gave more visually influenced responses than middle-aged males. Those middle-aged females consistently showed greater visual influence for AV speech could not simply be explained by gender differences in speech-reading proficiency coming to the fore as hearing acuity decreases. Differences in hearing levels between young and middle-aged adults were relatively small and both clearly inside the boundaries of normal hearing (British Society of Audiology, 2004). One may argue that a noisy background could reinforce the effect of such small differences in hearing acuity on auditory syllable perception (Baskent et al., 2014), but the AO performance in noise was similar between age groups. Most importantly, in calculating visual influence for AV speech, AO scores constituted the baseline for which the visual contribution to AV speech perception was measured.

For all groups, the percentage of AO syllables correctly identified in babble and white noise suggests that syllable identification could be substantially improved with visual speech cues available. Whereas these AO scores suggest that in noise all groups have similar perceptual incentive to shift toward more visually influenced responses, the results obtained for the AV stimuli show that middle-aged females gave considerably more visually influenced responses than the other groups. Whereas this special pattern of visual influence results for middle-aged females is difficult to explain by group differences in hearing acuity, AO performance, or in the noise induced perceptual incentive to make a shift toward more visual responses, the difference between young and middle-aged females may suggest that age-related AV experience may contribute to altering AV perceptual strategy. However, the lack of main effects of age in the contribution of visual cues in AV speech perception suggest that age-related changes in AV speech experience alone are insufficient to change the AV perceptual strategy. The study did not reveal main effects of age for speech-reading, AV benefit, or visual influence, even when the AV task difficulty was increased by noise. Whereas, the power analyses revealed that increasing the sample size might have rendered some of the small effects related to age significant, the means and effect sizes suggest that such small age effects are of negligible scientific interest. However, it must be stressed that power analyses may not be fully sensitive for a population that is known to be heterogeneous and that a comparatively larger sample size would be needed to ascertain the conclusions. Nevertheless, for visual influence in particular, such a small potential age difference seems to be a result of the performance of the middle-aged females exclusively, as the middle-aged males performed similarly to the young adults. The general conclusion related to age therefore remains that the age-related differences in the use of visual speech previously observed between young and older adults (over 60 years; e.g., Sommers et al., 2005; Winneke and Phillips, 2011; Sekiyama et al., 2014) are not present when comparing young and middle-aged adults (under 60 years). Hence, although the difference in visual influence observed between young and middle-aged females is in line with the notion that AV perceptual strategies may change in the course of a lifespan (e.g., Strelnikov et al., 2009) and that the AV perceptual strategy is sensitive to changes in AV speech experience (e.g., Strelnikov et al., 2009; Baskent et al., 2014; Huyse et al., 2014), the general lack of main effects for age in visual contribution to AV speech perception suggests that a critical prerequisite for change in AV perceptual strategy in middle-adulthood is a proficiency in speech-reading ability established at a younger age.

That the current study found gender differences in visual influence for middle-aged adults and replicated the frequently reported finding that females, independent of age, are better speech-readers than males (e.g., Johnson et al., 1988; Dancer et al., 1994; Watson et al., 1996; Strelnikov et al., 2009) suggests that although gender differences in speech-reading may be observed in young adulthood, age-related changes in AV experience may be needed for such visual proficiency to influence the general AV perceptual strategy. That gender differences in speech-reading are found for both age groups, whereas gender differences in visual influence are only obtained for middle-aged adults may indicate that increased visual reliance is an integral part of an experience dependent AV perceptual strategy, and to what degree one relies on the visual modality in middle adulthood may depend on one’s ability to reliably extract visual speech at an earlier age. Whereas the results for the young females indicate that proficiency in speech-reading does not automatically lead to greater reliance on visual speech cues in AV speech perception, the results for the middle-aged adults suggest that speech-reading proficiency may provide a conduit for AV speech experience such that recurrent confirmation of the contribution of visual speech cues may over time shift females’ AV perceptual strategies toward greater reliance on visual speech.

## Conflict of Interest Statement

The authors declare that the research was conducted in the absence of any commercial or financial relationships that could be construed as a potential conflict of interest.

## References

[B1] Alm M. Behne D. (2013). Audio-visual speech experience with age influences perceived audio-visual asynchrony in speech. *J. Acoust. Soc. Am.* 134 3001–3010. 10.1121/1.4820798 24116435

[B2] Alm M. Behne D. (2014). Age mitigates the correlation between cognitive processing speed and audio-visual asynchrony detection in speech. *J. Acoust. Soc. Am.* 136 2816–2826. 10.1121/1.4896464 25373981

[B3] Alm M. Behne D. M. Wang Y. Eg R. (2009). Audio-visual identification of place of articulation and voicing in white and babble noise. *J. Acoust. Soc. Am.* 126 377–387. 10.1121/1.3129508 19603894

[B4] Aloufy S. Lapidot M. Myslobodsky M. (1996). Differences in susceptibility to the “blending illusion” among native Hebrew and English speakers. *Brain Lang.* 53 51–57. 10.1006/brln.1996.0036 8722899

[B5] Baskent D. van Engelshoven S. Galvin J. J. III. (2014). Susceptibility to interference by music and speech maskers in middle-aged adults. *J. Acoust. Soc. Am.* 135 EL147–EL153. 10.1121/1.4865261 24606308 PMC4043475

[B6] Baxter L. Saykin A. Flashman L. Johnson S. Guerin S. Babcock D. (2003). Sex differences in semantic language processing: a functional MRI study. *Brain Lang.* 84 264–272. 10.1016/S0093-934X(02)00549-7 12590915

[B7] Benguerel A. P. Pichora-Fuller M. K. (1982). Coarticulation effects in lipreading. *J. Speech Hear. Res.* 25 600–607. 10.1044/jshr.2504.600 7162162

[B8] Berndl K. Dewitz W. Grüsser O. J. Kiefer R. H. (1986). A test movie to study elementary abilities in perception and recognition of mimic and gestural expression. *Eur. Arch. Psychiatry Neurol. Sci.* 235 276–281. 10.1007/BF00515914 3732338

[B9] Boersma P. Weenink D. (2009). *Praat: Doing Phonetics by Computer (Version 5.1) [Computer Program*]. Available at: http://www.praat.org/ [accessed February 8, 2009].

[B10] Bond Z. Moore T. (1994). A note on the acoustic-phonetic characteristics of inadvertently clear speech. *Speech Commun.* 14 325–337. 10.1016/0167-6393(94)90026-4

[B11] Bradlow A. Torretta G. Pisoni D. (1996). Intelligibility of normal speech I: global and fine-grained acoustic-phonetic talker characteristics. *Speech Commun.* 20 255–272. 10.1016/S0167-6393(96)00063-5 21461127 PMC3066472

[B12] British Society of Audiology. (2004). *Recommended Procedure: Pure Tone Air and Bone Conduction Threshold Audiometry with and Without Masking and Determination of Uncomfortable Loudness Levels.* Available at: http://www.thebsa.org.uk/docs/RecPro/PTA.pdf [accessed June 3, 2010].

[B13] Chen Y. Hazan V. (2009). Developmental factors and the non-native speaker effect in auditory-visual speech perception. *J. Acoust. Soc. Am.* 126 858–865. 10.1121/1.3158823 19640050

[B14] Clements A. Rimrodt S. Abel J. Blankner J. Mostofsky S. Pekar J. (2006). Sex differences in cerebral laterality of language and visuospatial processing. *Brain Lang.* 98 150–158. 10.1016/j.bandl.2006.04.007 16716389

[B15] Cohen J. (1988). *Statistical Power Analysis for the Behavioral Sciences* 2nd Edn. Hillsdale, NJ: Lawrence Erlbaum.

[B16] Dancer J. Krain M. Thompson C. Davis P. Glen J. (1994). A cross-sectional investigation of speechreading in adults: effects of age, gender, practice, and education. *Volta Rev.* 96 31–40.

[B17] Davis A. C. (1990). Epidemiological profile of hearing impairments: the scale and nature of the problem with special reference to the elderly. *Acta Otolaryngol.* 476 23–31. 10.3109/00016489109127252 2087969

[B18] Der G. Deary I. J. (2006). Age and sex differences in reaction time in adulthood: results from the United Kingdom health and lifestyle survey. *Psychol. Aging* 21 62–73. 10.1037/0882-7974.21.1.62 16594792

[B19] Dodd B. (1977). The role of vision in the perception of speech. *Perception* 6 31–40. 10.1068/p060031 840618

[B20] Easton R. D. Basala M. (1982). Perceptual dominance during lip reading. *Percept. Psychophys.* 32 562–570. 10.3758/BF03204211 7167355

[B21] Erber N. P. (1969). Interaction of audition and vision in the recognition of oral speech stimuli. *J. Speech Hear. Res.* 12 423–425. 10.1044/jshr.1202.423 5808871

[B22] Fixmer E. Hawkins S. (1998). “The influence of quality of information on the McGurk effect,” *Proceedings of the International Conference on Auditory-Visual Speech Processing* eds Burnham D. Robert-Ribes J. Vatikiotis-Bateson E. (Terrigal, NSW: AVSP) 27–32.

[B23] Foundas A. L. Leonard C. M. Hanna-Pladdy M. (2002). Variability in the anatomy of the planum temporale and posterior ascending ramus: do right and left-handers differ? *Brain Lang.* 83 403–424. 10.1016/S0093-934X(02)00509-6 12468396

[B24] Frost J. A. Binder J. R. Springer J. A. Hammeke T. A. Bellgowan P. S. Rao S. M. (1999). Language processing is strongly left lateralized in both sexes. Evidence from functional MRI. *Brain* 122 199–208. 10.1093/brain/122.2.199 10071049

[B25] Grant K. W. Seitz P. F. (1998). Measures of auditory-visual integration in nonsense syllables and sentences. *J. Acoust. Soc. Am.* 104 2438–2449. 10.1121/1.423751 10491705

[B26] Grant K. W. Walden B. E. Seitz P. F. (1998). Auditory–visual speech recognition by hearing-impaired subjects: consonant recognition, sentence recognition, and auditory–visual integration. *J. Acoust. Soc. Am.* 103 2677–2690. 10.1121/1.422788 9604361

[B27] Holm S. (1979). A simple sequentially rejective multiple test procedure. *Scand. J. Stat.* 6 65–70.

[B28] Hund-Georgiadis M. Lex U. Friederici A. D. von Cramon D. Y. (2002). Non-invasive regime for language lateralization in right- and left-handers by means of functional MRI and dichotic listening. *Exp. Brain Res.* 145 166–176. 10.1007/s00221-002-1090-0 12110956

[B29] Huyse A. Leybaert J. Berthommier F. (2014). Effects of aging on audio-visual speech integration. *J. Acoust. Soc. Am.* 136 1918–1931. 10.1121/1.4894685 25324091

[B30] Irwin J. R. Whalen D. H. Fowler C. A. (2006). A sex difference in visual influence on heard speech. *Percept. Psychophys.* 68 582–592. 10.3758/BF03208760 16933423

[B31] Jaeger J. Lockwood A. Van Valin R. D. Jr. Kemmerer D. L. Murphy B. W. Wack D. S. (1998). Sex differences in brain regions activated by grammatical and reading tasks. *Neuroreport* 9 2803–2807. 10.1097/00001756-199808240-00022 9760124

[B32] Johnson F. M. Hicks L. H. Goldberg T. Myslobodsky M. S. (1988). Sex differences in lipreading. *Bull. Psychon. Soc.* 26 106–108. 10.3758/BF03334875

[B33] Kass S. J. Ahlers R. H. Dugger M. (1998). Eliminating gender differences through practice in an applied visual spatial task. *Hum. Perform.* 11 337–349. 10.1207/s15327043hup1104_3

[B34] Kent R. D. (1997). *The Speech Sciences.* San Diego, CA: Singular Publishing Group 1–19.

[B35] Kimura D. (1992). Sex differences in the brain. *Sci. Am.* 267 119–125. 10.1038/scientificamerican0992-118 1298222

[B36] Kulynych J. J. Vladar K. Jones D. W. Weinberger D. R. (1994). Gender differences in the normal lateralization of the supratemporal cortex: MRI surface-rendering morphometry of Heschl’s gyrus and the planum temporal. *Cereb. Cortex* 4 107–118. 10.1093/cercor/4.2.107 8038562

[B37] Linn M. Petersen A. (1985). Emergence and characterization of sex differences in spatial ability. A meta-analysis. *Child Dev.* 39 102–116. 10.2307/1130467 4075870

[B38] Luchies C. W. Schiffman J. Richards L. G. Thompson M. R. Bazuin D. DeYoung A. J. (2002). Effects of age, step direction, and reaction condition on the ability to step quickly. *J. Gerontol. A Biol.* 57 M246–M249. 10.1093/gerona/57.4.M246 11909891

[B39] MacLeod A. Summerfield Q. (1987). Quantifying the contribution of vision to speech perception in noise. *Br. J. Audiol.* 21 131–141. 10.3109/03005368709077786 3594015

[B40] Markham D. Hazan V. (2004). The effect of talker- and listener-related factors on intelligibility for a real-word, open-set perception test. *J. Speech Lang. Hear. Res.* 47 725–737. 10.1044/1092-4388(2004/055) 15324282

[B41] McGee M. G. (1979). *Human Spatial Abilities: Sources of Sex Differences.* New York, NY: Praeger.

[B42] McGurk H. MacDonald J. (1976). Hearing lips and seeing voices. *Nature* (*London*) 264 746–748. 10.1038/264746a0 1012311

[B43] Miller G. A. Nicely P. E. (1955). An analysis of perceptual confusions among some English consonants. *J. Acoust. Soc. Am.* 27 338–352. 10.1121/1.1907526

[B44] Öhrström N. Traunmüller H. (2004). “Audiovisual perception of Swedish vowels with and without conflicting cues,” in *Proceedings of FONETIK 2004: The XVIIth Swedish Phonetics Conference* eds Branderud P. Traunmüller H. (Stockholm: Department of Linguistics) 40–43.

[B45] Parikh G. Loizou P. C. (2005). The influence of noise on vowel and consonant cues. *J. Acoust. Soc. Am.* 118 3874–3888. 10.1121/1.2118407 16419830

[B46] Pugh K. R. Shaywitz B. A. Shaiwitz S. E. Fulbright R. K. Byrd D. Skudlarski P. (1996). Auditory selective attention: an fMRI investigation. *Neuroimage* 4 159–173. 10.1006/nimg.1996.0067 9345506

[B47] Ross L. A. Saint-Amour D. Leavitt V. M. Javitt D. C. Foxe J. J. (2007). Do you see what I am saying? Exploring visual enhancement of speech comprehension in noisy environments. *Cereb. Cortex* 17 1147–1153. 10.1093/cercor/bhl024 16785256

[B48] Rouger J. Lagleyre S. Fraysse B. Deneve S. Deguine O. Barone P. (2007). Evidence that cochlear-implanted deaf patients are better multisensory integrators. *Proc. Natl. Acad. Sci. U.S.A.* 104 7295–7300. 10.1073/pnas.0609419104 17404220 PMC1855404

[B49] Ruytjens L. Albers F. van Dijk P. Wit H. Willemsen A. (2006). Neural responses to silent lipreading in normal hearing male and female subjects. *Eur. J. Neurosci.* 24 1835–1844. 10.1111/j.1460-9568.2006.05072.x 17004947

[B50] Ruytjens L. Georgiadis J. R. Holstege G. Wit H. P. Albers F. W. Willemsen A. T. (2007). Functional sex differences in human primary auditory cortex. *Eur. J. Nucl. Med. Mol. Imaging* 34 2073–2081. 10.1007/s00259-007-0517-z 17703299 PMC2100432

[B51] Sekiyama K. Burnham D. Tam H. Erdener D. (2003). “Auditory-visual speech perception development in Japanese and English speakers,” in *Proceedings of the International Conference on Auditory-Visual Speech Processing* (St. Jorioz: AVSP) 61–66.

[B52] Sekiyama K. Soshi T. Sakamoto S. (2014). Enhanced audiovisual integration with aging in speech perception: a hightened McGurk effect in older adults. *Front. Psychol.* 5:323 10.3389/fpsyg.2014.00323 PMC3995044 24782815

[B53] Sekiyama K. Tohkura Y. (1993). Inter-language differences in the influence of visual cues in speech perception. *J. Phonetics* 21 427–444.

[B54] Shaywitz B. A. Shaywitz S. E. Pugh K. R. Constable R. T. Skudlarski P. Fulbright R. K. (1995). Sex differences in the functional organization of the brain for language. *Nature* 373 607–609. 10.1038/373607a0 7854416

[B55] Sommer I. E. Aleman A. Bouma A. Kahn R. S. (2004). Do women really have more bilateral language representation than men? A meta-analysis of functional imaging studies. *Brain* 127 1845–1852. 10.1093/brain/awh207 15240433

[B56] Sommers M. S. Tye-Murray N. Spehar B. (2005). Auditory-visual speech perception and auditory-visual speech enhancement in normal-hearing younger and older adults. *Ear Hear.* 26 263–275. 10.1097/00003446-200506000-00003 15937408

[B57] Strelnikov K. Rouger J. Lagleyre S. Fraysse B. Deguine O. Barone P. (2009). Improvement in speech-reading ability by auditory training: evidence from gender differences in normally hearing, deaf and cochlear implanted subjects. *Neuropsychologia* 47 972–979. 10.1016/j.neuropsychologia.2008.10.017 19022268

[B58] Sumby W. H. Pollack I. (1954). Visual contribution to speech intelligibility in noise. *J. Acoust. Soc. Am.* 26 212–215. 10.1121/1.1907309

[B59] Summerfield Q. (1992). Lipreading and audio–visual speech perception. *Philos. Trans. R. Soc. Lond. B Biol. Sci.* 335 71–78. 10.1098/rstb.1992.0009 1348140

[B60] Suresh K. P. Chandrashekara S. (2012). Sample size estimation and power analysis for clinical research studies. *J. Hum. Reprod. Sci.* 5 7–13. 10.4103/0974-1208.97779 22870008 PMC3409926

[B61] Traunmüller H. Öhrström N. (2007). Audiovisual perception of openness and lip rounding in front vowels. *J. Phonetics* 35 244–258. 10.1016/j.wocn.2006.03.002

[B62] Tye-Murray N. Sommers M. Spehar B. (2007). The effects of age and gender on lipreading abilities. *J. Am. Acad. Audiol.* 18 883–892. 10.3766/jaaa.18.10.7 18496997

[B63] Tyler R. S. Parkinson A. J. Woodworth G. G. Lowder M. W. Gantz B. J. (1997). Performance over time of adult patients using the Ineraid or Nucleus cochlear implant. *J. Acoust. Soc. Am.* 102 508–522. 10.1121/1.419724 9228814

[B64] Voyer D. Voyer S. Bryden M. P. (1995). Magnitude of sex differences in spatial abilities: a meta-analysis and consideration of critical variables. *Psychol. Bull.* 117 250–270. 10.1037/0033-2909.117.2.250 7724690

[B65] Walden B. E. Prosek R. A. Montgomery A. A. (1977). Effects of training on the visual recognition of consonants. *J. Speech Lang. Hear. Res.* 20 130–145. 10.1044/jshr.2001.130 846196

[B66] Walla P. Hufnagl B. Lindinger G. Deecke L. Lang W. (2001). Physiological evidence of gender differences in word recognition: a magnetoencephalographic (MEG) study. *Cogn. Brain Res.* 12 49–54. 10.1016/S0926-6410(01)00028-3 11489608

[B67] Watson C. S. Qiu W. W. Chamberlain M. M. Li X. (1996). Auditory and visual speech perception: confirmation of a modality-independent source of individual differences in speech recognition. *J. Acoust. Soc. Am.* 100 1153–1162. 10.1121/1.416300 8759968

[B68] Winneke A. H. Phillips N. A. (2011). Does audiovisual speech offer a fountain of youth for old ears? An event-related brain potential study of age differences in audiovisual speech perception. *Psychol. Aging* 26 427–438. 10.1037/a0021683 21443357

